# Supplementary material for: Diffraction-engineered holography: Beyond the depth representation limit of holographic displays
Source: Nat Commun. 2022 Oct 12;13:6012. doi: 10.1038/s41467-022-33728-5 (PMC9556550; doi:10.1038/s41467-022-33728-5)
Supplement: Supplementary file 1 — Supplementary Information [file 41467_2022_33728_MOESM1_ESM.pdf]

Supplementary Material

1 Phase noise of the amplitude-only SLM

For a given amplitude modulation, $f(x)$, and a corresponding unwanted phase modulation depending on the amplitude modulation, $g(f(x))$, we can approximate $g(f(x))$ with Tayler Series expansion.

$$e^{ig(f(x))} = e^{ig(0)+ig'(0)f(x)+ig''(0)f(x)^2+\mathcal{O}(f(x)^3)} \tag{S1}$$

Here, we used only upto a second order, since the second order was empirically enough. To prove the fact, we measured relative phase depending on its amplitude (Fig. S1). While blocking most of the LCoS, only two sections are exposed to input collimated laser and interference of modulated amplitude reflected from the two sections is measured. By sweeping intensity of one section, while the intensity of the other section remains to the brightest value, relative phase can be estimated from the interference pattern. At the sensor, the interference signal between two arbitrary phase light can be calculated as,

$$\begin{aligned} &\left| e^{-2r^2/w^2}\sqrt{I_1}e^{ik_1x+i\phi_1(I_1)} + e^{-2r^2/w^2}\sqrt{I_2}e^{ik_2x+i\phi_2(I_2)}\right|^2 \\ &= e^{-2r^2/w^2}\left[I_1 + I_2 + 2\sqrt{I_1I_2}\cos((k_2-k_1)x+\phi_2(I_1)-\phi_1(I_2))\right], \end{aligned} \tag{S2}$$

Here, we assumed that the two beams are perfectly aligned and the propagation distance is large enough. If a propagation distance of arbitrary coherent light is large enough, then the envelope shape of the beam becomes Gaussian. Moreover, geometric factors of phase including radius of curvature and Gouy phase remain unchanged under the intensity sweeping, so the relative phase $\phi_2(I_1)-\phi_1(I_2)$ in Eq. S2 indicates the unwanted phase oriented from the intensity.

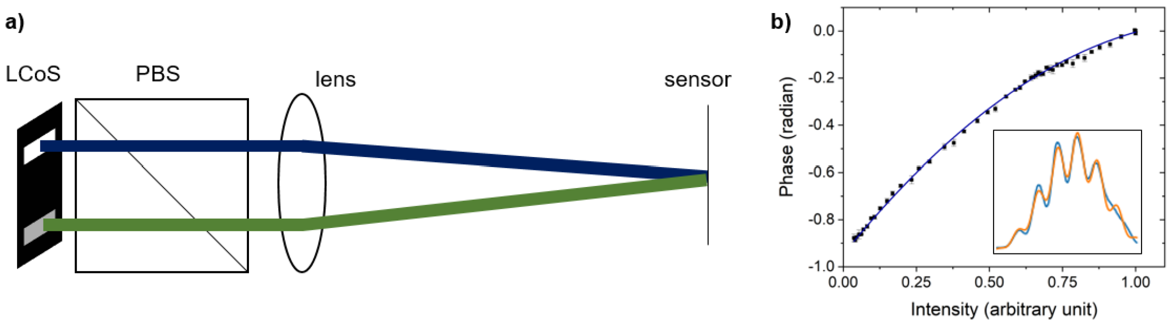

Figure S1: **Schematics of experimental setup for intensity-phase measurement and the result. a,** Experimental setup for intensity-phase measurement **b,** Result of phase-ampltiude measurement. Intensity is normalized to a maximum intensity and phase is given in a unit of radian. Black dots are averages of measured data for each phase and error bars indicate standard deviation of independent measurements. Blue line is second order polynomial fit of the data. Blue line in inset represents interference pattern at the sensor and red line in inset represents fitted curve using Eq. S2.

By fitting the Eq. S2, we could extract the relative phase between two different amplitude beams. Figure S1**b** shows the measured data for each intensity and the second order polynomial fit of the data. As we can see here, unwanted phase modulation can be approximated as Eq. S1 at least for our experimental setup.

2 Comparison with the other algorithm

In order to explicitly depict the differences between our algorithm and tensor holography algorithm [1], we compared the holograms by simulation (Fig. S2). While synthesizing the hologram of tensor holography, input depth map is rescaled because the network of tensor holography covers different depth range from DEHNet. After synthesizing the holograms, simulated focus is tuned from front to back and each reconstructed intensity is compared with the rendered image of that focus.

Although tensor holography gives high PSNR for the in-focus object according to the re-

search [1] ($>$ 30 dB), image quality of the reconstructed intensity at some focal plane is far lower than that of DEHNet since the intensity of out-of-focus objects is not considered while evaluating image quality in the tensor holography. By evaluating PSNR(SSIM) of the reconstructed holograms for different 21 focal planes with respect to the corresponding rendered images, PSNR(SSIM) of DEHNet result is higher than that of tensor holography 3.8 dB (0.11) on average for the scene used in Fig. S2. Especially for the last column of Fig. S2, the amount of defocus blur shows huge difference. Indeed, the result of the tensor holography is similar to that of the nCGH, since the tensor holography algorithm is based on the nCGH.

Furthermore, we also compared the reconstructed results of dCGH which utilizes random phase to diffuse light [2]. Due to the random phase, image quality metrics are much lower than other algorithms. However, if time-multiplexing method is simultaneously adopted, then image quality is increased and defocus blur becomes comparable to the that of rendered images.

Table S1 shows summarized benchmark results of different holograms evaluated on the FHD resolution dataset. DEH, DEHNet(fp32), and DEHNet(fp16) show best image quality metrics while DEHNet offers fastest synthesis speed.

3 Effect of diffuser

A diffuser is used to reduce the ringing pattern originated from the dust at lenses. Since the area of our SLM only 13.8×7.8 mm^2, usual dust (1$\sim$100 μm) can block significant area of the wavefield. Without a diffuser, the wavefield modulated by a pixel of SLM behaves like a single ray and generates noise near the dust if the single ray is blocked. Recent research successfully suppressed such noise using camera-in-the-loop algorithm [5], but even the algorithm cannot fully suppress noise if the spatial coherence length is infinity. In order to slightly reduce the spatial coherence length, LEDs or lasers with an optical diffuser can be used. Figure S3 shows

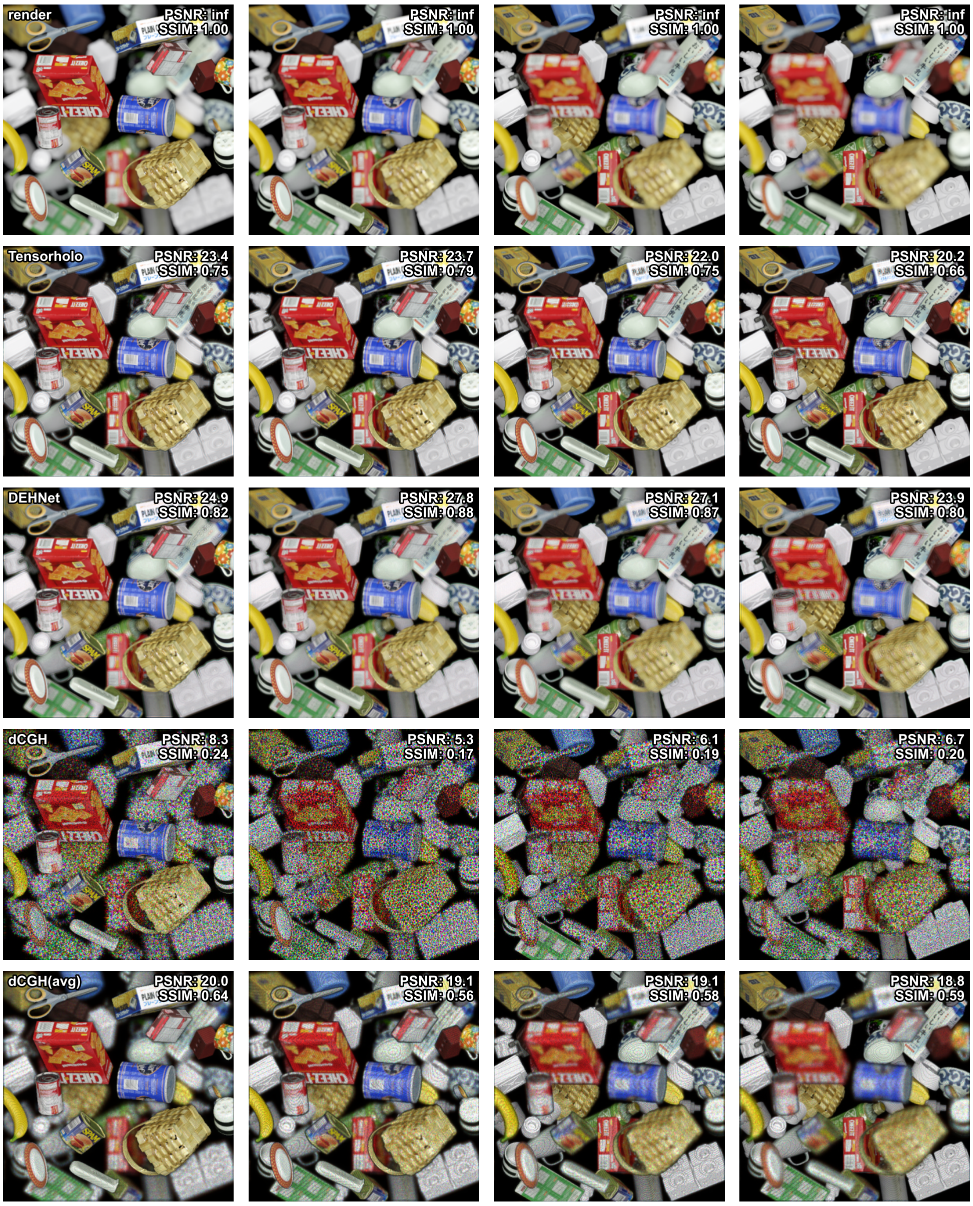

Figure S2: **Comparison with the other algorithm.** Intensities of the holograms are reconstructed by simulations with varying their focal planes. Focal planes of the represented images correspond to 0.15, 1.05, 1.95, and 2.85 diopter(from left column to right column). PSNR and SSIM are evaluated with respect to the rendered image located the same focal plane. “render” refers rendered images, “DEHNet” refers simulated intensity of DEHNet result, “Tensorholo” refers simulated intensity of tensor holography [1], “dCGH” refers simulated intensity of diffusive hologram [2], and “dCGH(avg)” refers average image of dCGHs with 100 different random phases.

Type	PSNR (dB)	SSIM	vgg16	alex	Time (s)
DEH	33.4±0.61	0.955±0.0039	0.0743±0.0048	0.0415±0.0038	137±0.024
DEHNet(fp32)	33.4±0.57	0.957±0.0035	0.0778±0.0047	0.0427±0.0037	$0.0609\pm 2.7\cdot10^{-5}$
DEHNet(fp16)	33.4±0.57	0.956±0.0035	0.0778±0.0047	0.0427±0.0037	$0.0287\pm 4.1\cdot10^{-5}$
DEHNet	32.7±0.57	0.939±0.0040	0.126±0.0080	0.0542±0.0039	$0.0159\pm 1.8\cdot10^{-4}$
nCGH	27.1±0.59	0.889±0.0079	0.166±0.0088	0.136±0.0078	1.11±0.047
Tensorholo(fp16)	29.9±0.65	0.918±0.0063	0.124±0.0062	0.0898±0.0058	0.0403±0.0011
dCGH	8.06±0.59	0.306±0.0350	0.637±0.028	0.911±0.041	1.71±0.0033

Table S1: **Benchmark of different holograms.** DEHNet(fp32) refers DEHNet before quantization, DEHNet(fp16) refers DEHNet quantized to float16 data type, and Tensorholo(fp16) refers the float16 quantized version of the pre-trained network[1]. For the metrics, vgg16(alex) refer learned perceptual metrics calculated using vgg-16(alex) loss [3, 4]. Lower is better for learned perceptual metrics. Tensorholo(fp16) is quantized to float16 data type by using TensorRT library. Averages of metrics are evaluated on FHD resolution dataset and their standard deviations presents standard deviations between scenes in the dataset.

the optical reconstruction result of the cases with a diffuser and without a diffuser.

4 Projection of SLM

Synthesis of our hologram is based on orthographic projection and adopts equally spaced focal planes, whereas the scene perceived by an eye is rather similar to perspective projection. Moreover, amount of defocus blur of an eye is proportional to a diopter of crystalline lens, while amount of defocus blur of the synthesized hologram is proportional to the propagation distance. The gap between the configurations can be explained by magnification power of the objective lens (Fig. S4). By placing the SLM near the focal plane of the objective lens, each sampling plane is magnified and forms virtual image at the distance f',

$$f' = \frac{f(f + \Delta z_i)}{\Delta z_i} \approx \frac{f^2}{\Delta z_i}. \tag{S3}$$

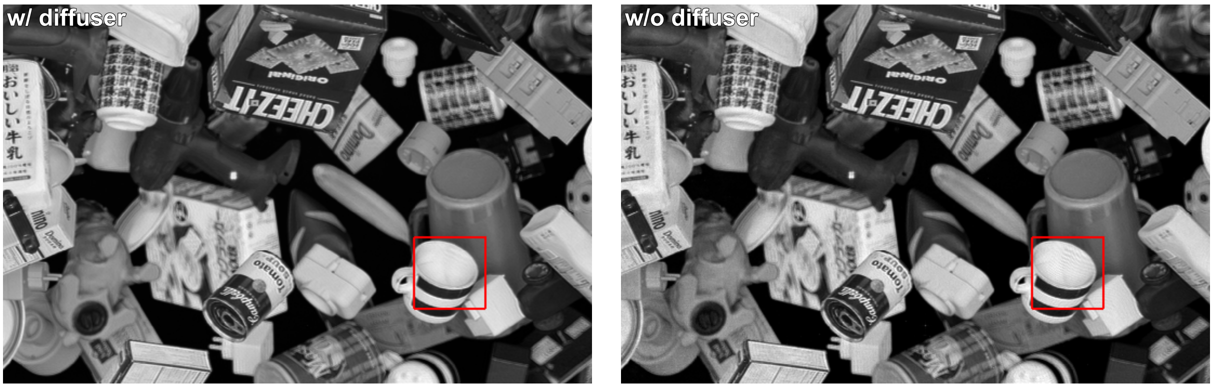

Figure S3: **Experiments with and without diffuser.** Here, only green laser (wavelength 520 nm) is used in both cases. Although they are not noticeably different, there exist ringing pattern near the zebra cup(red rectangle) in the experiment without diffuser.

Here, f is the effective focal length of the objective lens, Δz_i is the distance between the ith sampling plane and the focal plane of the objective lens, and $\Delta z_i \ll f$ is assumed.

Considering the diopter of crystalline lens is defined as $1/f' \approx \Delta z_i/f^2$, defocus blur of an eye is linearly proportional to Δz_i as the defocus blur of the synthesizing targets. Furthermore, virtual images of sampling planes are magnified and the series of the planes can be seen as a perspective view. Under this projection schematics, if the content with perspective view is used then the scene is same with the scene perceived by an eye.

5 Corner case

Since our textures of the training dataset and those of the evaluation dataset are completely different and thus the general performance of our model is expected to be around the performance evaluated on the evaluation dataset. One of the possible exceptions would be a scene with sparsely placed objects since the dataset is consisted of the scenes with densely placed objects. In order to prove the generality of the trained neural networks in that case, sparse scene is evaluated in Fig. S5.

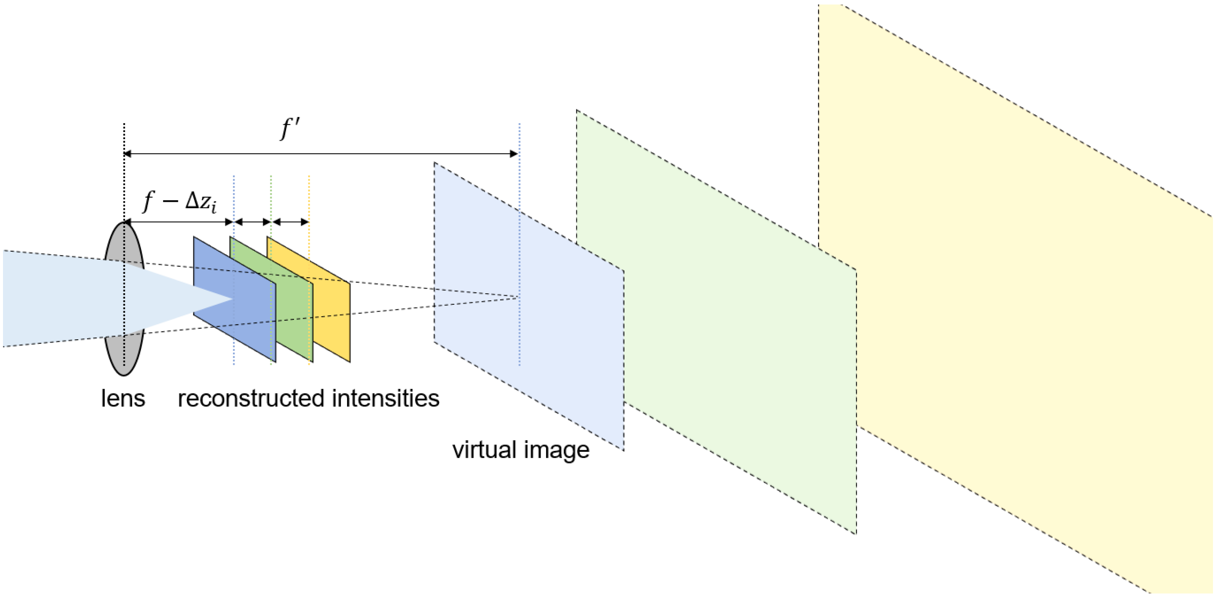

Figure S4: **Schematics of SLM projection.** When SLM is placed near the focal point of objective lens, the virtual image of the SLM is created near the infinity. As Δz_i increases, the location of the virtual image becomes closer and magnification becomes smaller. Solid parallelogram indicates each sampling plane of DEHNet and dotted parallelogram indicates each virtual image projected by the objective lens. Here, collimated laser is assumed as input light.

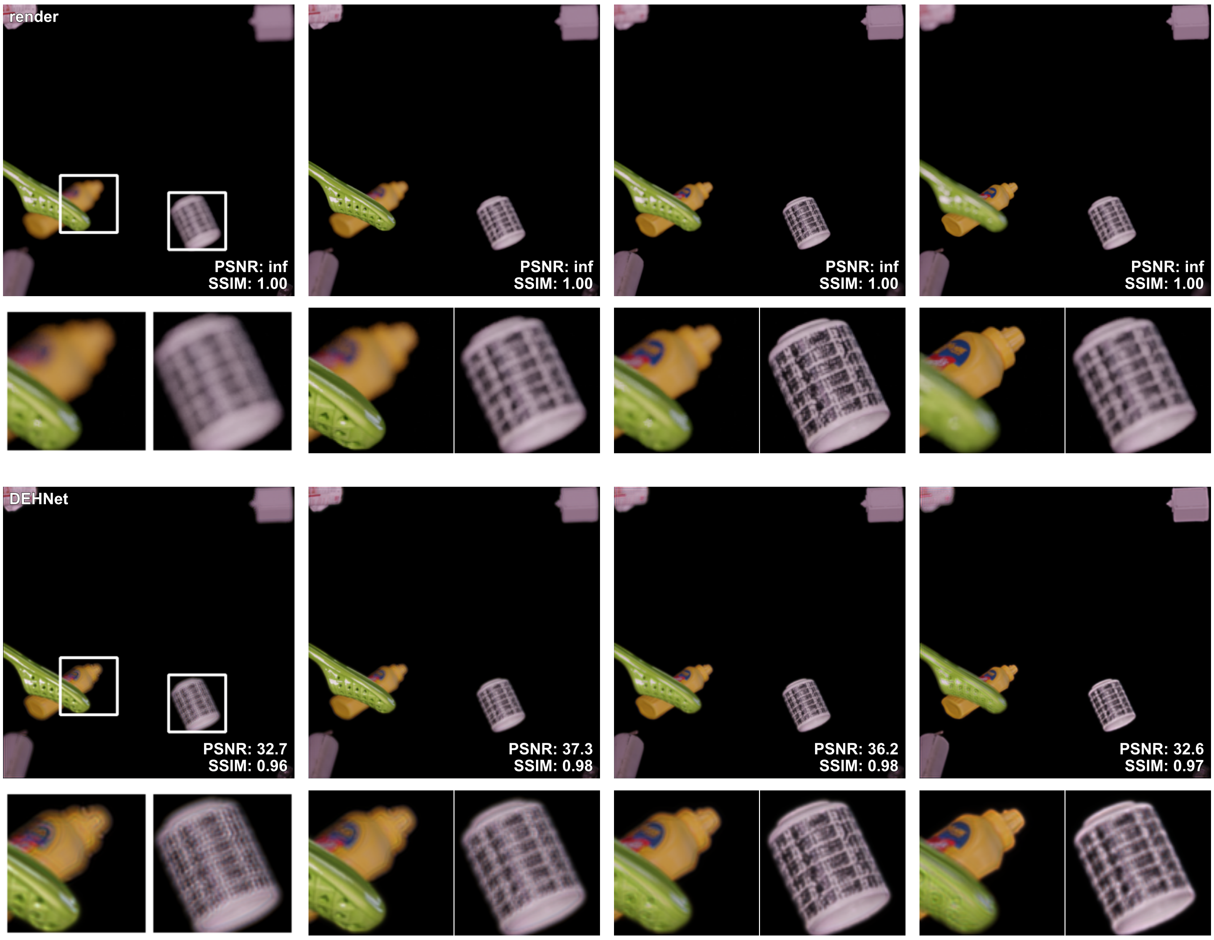

Figure S5: **Scene with sparsely placed objects.** Hologram of the scene with sparsely placed objects is synthesized by DEHNet and their image quality metrics are evaluated by simulation. Focal planes of the represented images correspond to 0.15, 1.05, 1.95, and 2.85 diopter(from left column to right column). “render” refers rendered images and “DEHNet” refers simulated intensity of DEHNet result.

Not surprisingly, image quality is sustained high enough for the shown case. Indeed, this behavior can be expected from the receptive field and the number of the parameters of our model. Because receptive field of our network is 69 pixels $\times$ 69 pixels, if the distance between pixels is larger than 69, their calculated values are independent. Moreover, the number of the parameters of our model is only 45k and the small number prevents our model from overfitting to a specific scene(Unet: 30M, Resnet18: 11M, Resnet50: 24M).

6 Requirements for the number of target planes

Defocus blur is induced by propagation of incoherent light and thus the diameter of defocus blur is proportional to the distance between the object and the focal plane. If a distance between two nearest individual planes corresponds to the distance producing one-pixel-diameter of defocus blur, then the intensity shift by propagation of incoherent light between the two nearest individual planes would be 1 pixel. In order to set enough target images for pixel-level accuracy, we set the number of individual planes to the number which is larger than the maximum diameter of the scene.

In this sense, the number of individual planes depends on the maximum defocus blur derived from the maximum depth of scene we want to reconstruct. In the manuscript, maximum diameter of blur circle is 15 pixels when the maximum depth of presented scenes is 3 diopter (Please see "Determining the diameter of the blur circle section" of Methods). To ensure pixel-level accuracy, the number of planes are selected as 21, larger than the maximum diameter of the blur circle. In the same way, if the maximum depth is selected as 5 diopter, then at least 25 planes are required to ensure pixel-level accuracy.

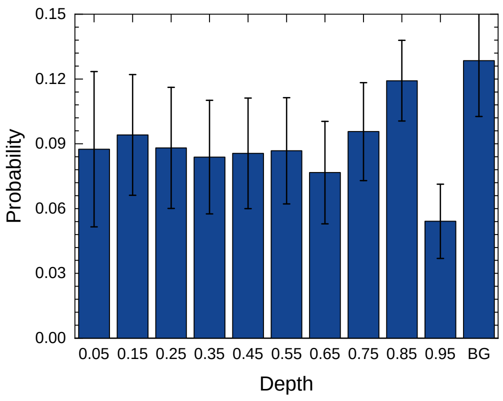

Figure S6: **Average depth distribution of training dataset.** Each bar represents depth probability between the range $[x - 0.05, x + 0.05)$, where x refers a labeled value. The depth value 0 corresponds to the minimum depth and the depth value 1 corresponds to the maximum depth. BG refers background of scenes, corresponding to the maximum depth value. Error bar represents standard deviation of each distribution between scenes.

7 Depth distribution of training dataset

To clearly present the effect of the DEH, we selected one of rendered images of which depth difference between objects are significant in the figures of the manuscript. The depth distribution of training dataset is more uniform than the figures in the manuscript to prevent the model from overfitting to a particular depth during training. Fig. S6 presents average depth distribution of all scenes in the training dataset. Depth distribution near the maximum depth is minimized to overlap between objects and the background. To accomplish such distribution, objects are moved before rendering scenes if some depth range is too crowded. During re-distributing the objects, we divided the whole depth range into 5 ranges and moved 3 objects at the depth with maximum probability if the maximum depth probability is 20% larger than the minimum depth probability. Figure S7 presents examples image and depth maps of different datasets.

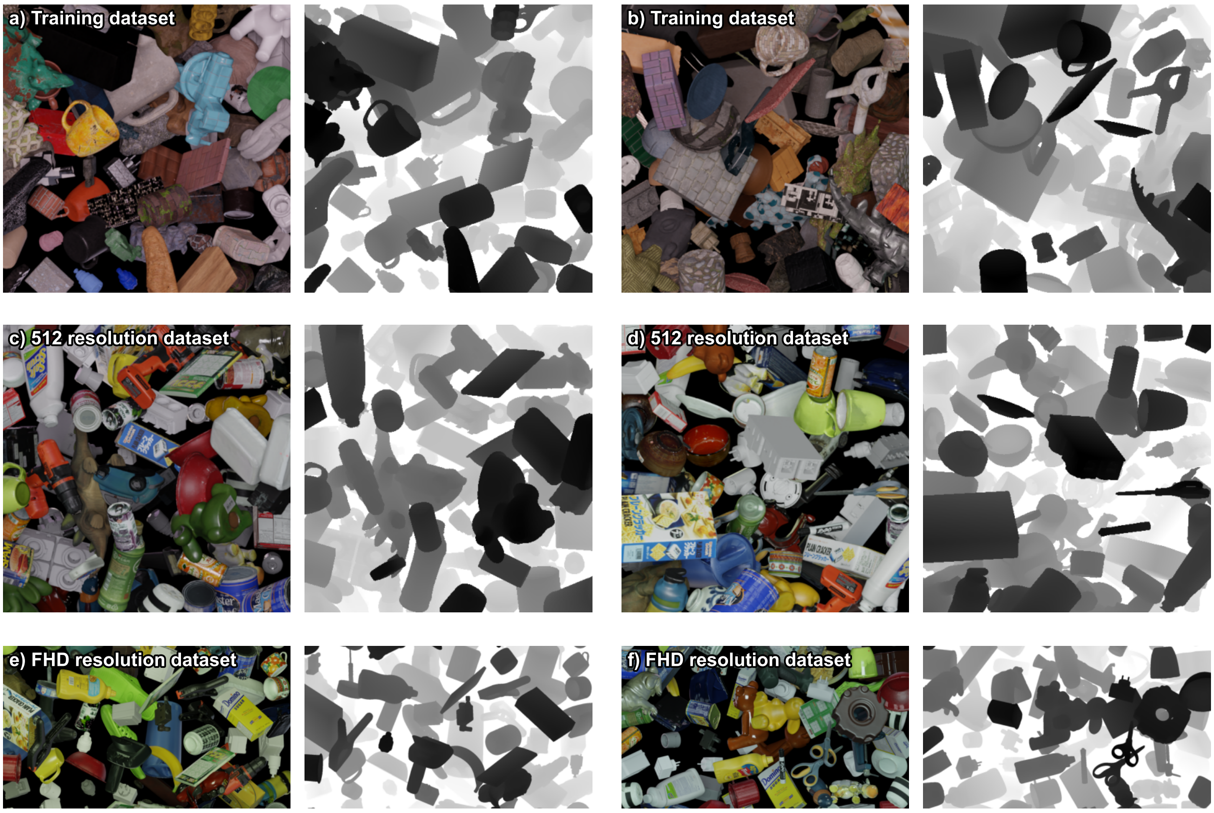

Figure S7: **Example all-in-focus images and depth maps of dataset. a, b,** All-in-focus images and depth maps of training dataset. **c, d,** All-in-focus images and depth maps of 512 resolution evaluation dataset. **e, f,** All-in-focus images and depth maps of FHD resolution evaluation dataset.

Type	PSNR (dB)	SSIM	vgg16	alex	GFLOPS
c48	32.3±0.94	0.943±0.010	0.0864±0.0072	0.0523±0.0074	396.8
c24	33.3±0.24	0.955±0.0018	0.0790±0.0021	0.0436±0.0017	187.9
c12	33.1±0.10	0.954±0.00072	0.0811±0.00066	0.0458±0.00062	91.5
c6	32.8±0.11	0.950±0.00088	0.0850±0.00094	0.0494±0.00059	46.6

Table S2: **Image quality depending on the structures of the models.** c48(c24, c12, c6) refers the model with 48(24, 12, 6) channels and 10(18, 34, 68) hidden layers. FLOPS refers floating-point operations per second. The metrics are evaluated on the FHD resolution dataset. Average metrics and its standard deviation are calculated using multiple trained models with same condition. Among 4 trained models, one with the worst PSNR is excluded and 3 models are used in averaging. The mean metrics of c12 is different from the metrics of Fig. 5 because we selected the best model among c12 models.

8 Image quality dependency on number of channels

In the manuscript, we used the neural network model with 12 channels and 34 hidden layers. Table S2 presents summarized image quality depending on the structures of the models. Although the structures of networks do not much change image quality, c24 and c12 models show better image quality than other models. Since 6 is the minimum number of channels to express complex 3-color field, numbers of channels 24 and 12 is not much larger than the minimum value.

An architecture of our model is repeat of the unit structure, Conv2d - PReLU - Conv2d - PReLU with identity shortcut, except for the first unit. For the first unit, shortcut is Conv2d layer with kernel size 1 and the number of channels in Conv2d layer is changed from the number of input channels (4) to the number of output channels (12). After concatenating the result of the last layer and the input layer, the channel size is reduced to 6 by a Conv2d layer with kernel size 3.

9 Effect of all-in-focus loss

We adopted all-in-focus loss in Eq. 2 in order to reconstruct sharply focused objects. The effect of the loss can be quantified by comparing image qualities of reconstructed intensities at target planes and pixel-wise reconstruction accuracy at each depth of each pixel compared to an all-in-focus image. The former metric is calculated as the first term in Eq. 2 and it evaluates image qualities of defocus blur and focused images. The latter metric is calculated by propagating the hologram for every unique depth of input depth map and comparing the pixels at that depth with an all-in-focus image, where the depth values in input depth map can be 256 different values. As a result, the latter metric only represents focused image quality.

Figure S8 presents two different metrics, so called varifocal PSNR and all-in-focus(AIF) PSNR. After training the model with varying β, we evaluated the metrics on the FHD resolution dataset. As β parameter increases, AIF PSNR increases and varifocal PSNR decreases. As we expected, varifocal PSNR and AIF PSNR becomes close at the point $\beta = 20$.

However, AIF PSNR can be inaccurate when the defocus blur comes into the image, so we do not used the metric in the manuscript. For instance, when the focal plane is at the rear plane, a rear object is occluded by defocus blur of front object. However, blurred front object is bigger than the original size of all-in-focus image due to defocus blur. As a result, even if the rear object is in focus, it is more natural to have different values from the all-in-focus image for some part of the rear object at the defocus blur of the front object. Such incorrect values are common when multiple objects are located at different depths and the circumstance is close to our rendered scenes. (Please see Fig. 9 and related explanations)

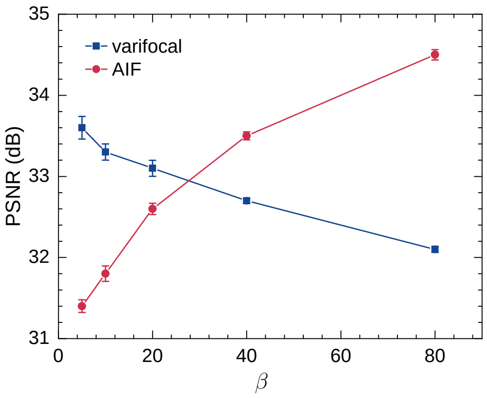

Figure S8: **Beta dependency of varifocal PSNR and AIF PSNR.** Blue square represents varifocal PSNR and red circle represents AIF PSNR. Error bars represent standard deviations of models trained with the same conditions. Among 4 trained models for each condition, one with the worst PSNR is excluded and 3 models are used in averaging.

10 Effect of occluded surface in hologram

In a 3D scene, occluded surface affects the edge of defocus blur of a front object when the focal plane is at the rear side. Light from the occluded surface of a rear object partially participates in the defocus blur of the front object. However, there is no information of the occluded surfaces in the pinhole RGBD image and thus partial participation of occluded object is hard to consider in hologram synthesis.

Figure S9 presents how the RGBD pinhole image losses information of the occluded surface and its effect on the reconstructed scene. Due to the lack of information, the occluded surface is usually assumed to be empty during the synthesis of nCGH and so the line near the edge is displayed with background color (*i.e.* black) at the defocus blur of the front object. In contrast, if we use the whole information of the scene by including the complete red circle during the nCGH synthesis, the edge line diminishes. For the reconstructed image of DEHNet, there is no black line near the edge of the front object. From the fact, it seems like DEHNet utilizes information

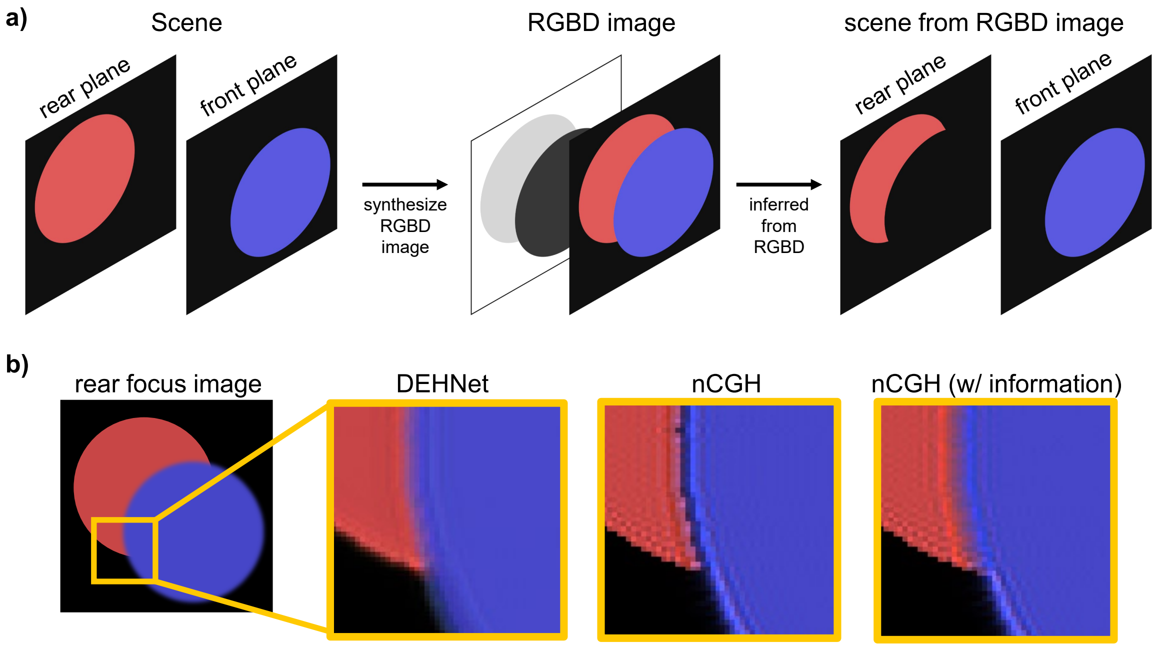

Figure S9: **Information loss of occluded surface and its effect on hologram.** **a**, Information loss of occluded surface. Scene refers target 3D scene of the hologram. **b**, Effect of information loss on reconstructed rear focus image of holograms. "nCGH (w/ information)" refers reconstructed nCGH with using full information of the rear plane, *i.e.* complete red circle.

near the occluded surface and remove such edge line although the detailed mechanism is hard to confirm.

11 Maximum scene depth of DEH

Although maximum scene depth under the current parameter is 3 diopter, the maximum scene depth can be arbitrarily increased. The side effect of increasing the maximum scene depth is decreased image quality since the number of individual planes that hologram should reconstruct increases. To evaluate image quality metrics depending on the maximum scene depth, we synthesized 200 scenes with maximum scene depth 9 diopters and 61 focal planes. The number of planes per diopter was set to 6.67 planes/diopter as in the manuscript and analyzed image

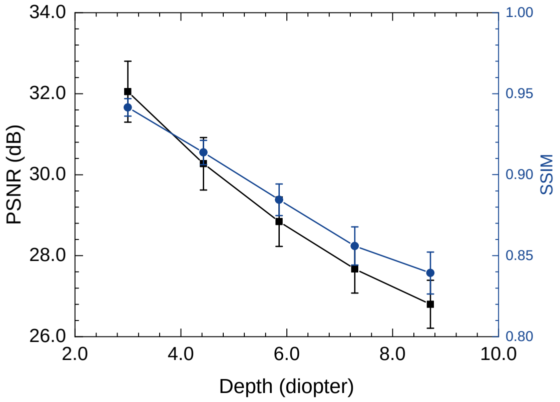

Figure S10: **Image quality metrics depending on maximum scene depth.** Black square (blue circle) represents PSNR in dB (SSIM) and values are marked on the left (right) axis. Error bar indicates standard deviation between scenes.

quality depending on the maximum scene depth using optimization method to avoid the effect coming from capability of a neural network. The 61 plane dataset was synthesized using the same method as the previous dataset and its resolution was FHD.

When optimizing and evaluating the hologram with smaller maximum scene depth than 9 diopter, subset of 61 planes are used. For instance, 31 planes are used for optimizing and evaluating DEH with 4.5 diopter, and 41 planes are used for 6 diopter DEH. By using the same scenes in evaluating the DEHs possessing different maximum depths, it is possible to evaluate relative difference between them. Figure S10 presents image quality metrics depending on maximum scene depth. As maximum scene depth increases image quality metrics decreases. If we set the image quality criteria as PSNR $>$ 30 dB, then 4.5 diopter is the maximum scene depth we can use. However, the criteria can be arbitrarily selected and someone may use DEH with 6.0 diopter if the image quality is not the top priority.

12 Optimization with perceptual loss

According to the research [6], employment of perceptual loss in optimization improves image quality of reconstructed holograms. Since multi-scale structural similarity (MS-SSIM) loss gives the best image quality among different losses, we adopted MS-SSIM loss to optimize DEH. Figure S11 presents experimental results of the DEH optimized using MS-SSIM loss. First term of Eq. 2 is replaced with MS-SSIM loss and β is increased until the all-in-focus PSNR gets similar to varifocal PSNR (see Section "Effect of all-in-focus loss").

Although MS-SSIM loss gives the best image quality in single plane holograms, DEH optimized by MS-SSIM loss suffers from some defects. For instance, defocus blur seems inappropriate at the letters of a can in the MS-SSIM result (enlarged image of rear focus in Fig. S11). In contrast to single plane holograms, which were investigated in the research [6], further investigation of loss functions could be another research topic for multi-plane holograms.

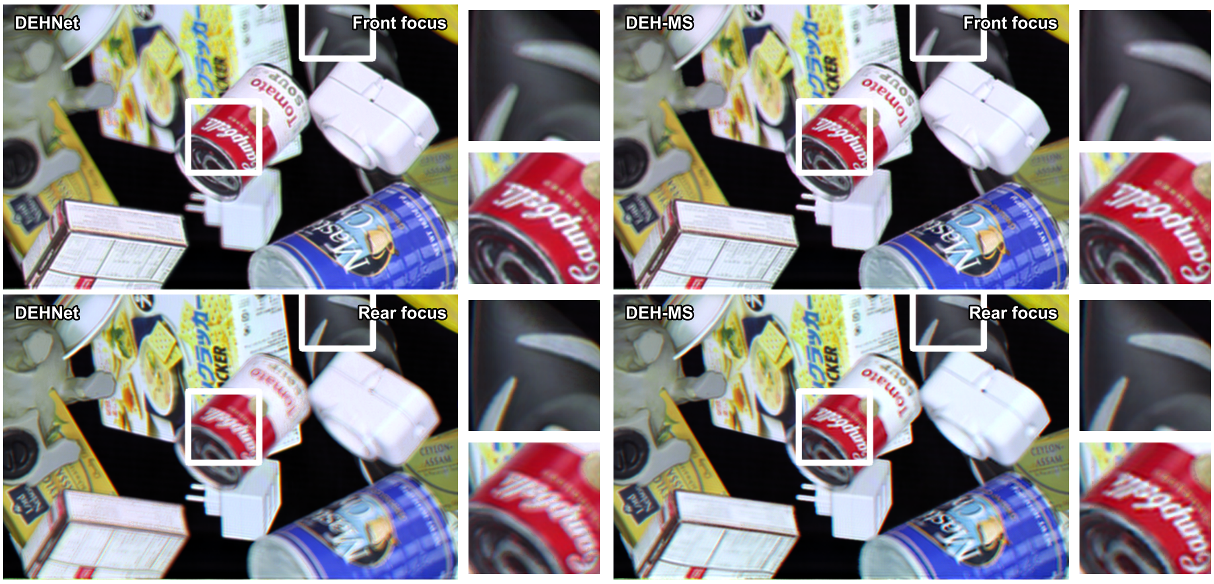

Figure S11: **Experimental results of the DEH optimized by MS-SSIM loss.** The top images correspond to the front focus images and the bottom images correspond to the rear focus images. The small images represent enlargements of the corresponding reconstructions, as indicated by the white squares. DEH-MS refers the reconstructed image of the DEH optimized using MS-SSIM loss.

References

1. Shi, L., Li, B., Kim, C., Kellnhofer, P. & Matusik, W. Towards real-time photorealistic 3D holography with deep neural networks. *Nature* **591,** 234–239 (2021).

2. Zhao, Y., Cao, L., Zhang, H., Kong, D. & Jin, G. Accurate calculation of computer-generated holograms using angular-spectrum layer-oriented method. *Optics Express* **23,** 25440–25449 (2015).

3. Ledig, C. *et al. Photo-realistic single image super-resolution using a generative adversarial network* in *Proceedings of the IEEE conference on computer vision and pattern recognition* (2017), 4681–4690.

4. Zhang, R., Isola, P., Efros, A. A., Shechtman, E. & Wang, O. *The unreasonable effectiveness of deep features as a perceptual metric* in *Proceedings of the IEEE conference on computer vision and pattern recognition* (2018), 586–595.

5. Peng, Y., Choi, S., Padmanaban, N. & Wetzstein, G. Neural holography with camera-in-the-loop training. *ACM Transactions on Graphics (TOG)* **39,** 1–14 (2020).

6. Yang, F. *et al.* Perceptually motivated loss functions for computer generated holographic displays. *Scientific reports* **12,** 1–12 (2022).
