# Supplementary material for: Diffraction-engineered holography: Beyond the depth representation limit of holographic displays
Source: Nat Commun. 2022 Oct 12;13:6012. doi: 10.1038/s41467-022-33728-5 (PMC9556550; doi:10.1038/s41467-022-33728-5)
Supplement: Supplementary file 3 — Description to Additional Supplementary Information [file 41467_2022_33728_MOESM3_ESM.pdf]

Description of Additional Supplementary Files

Supplementary Movie 1

Animation of the numerical reconstruction of a scene with changing the focal plane.

Supplementary Movie 2

Animation of the experimental reconstruction of a scene with changing the focal plane.
